# Beyond stigma: integrating social and diagnostic determinants of delayed leprosy detection^؆^

**DOI:** 10.1016/j.abd.2026.501460

**Published:** 2026-08-29

**Authors:** Patrícia Tavares Cruz, Hélio Amante Miot, Carolina Talhari, Sinésio Talhari

**Affiliations:** a Department of Teaching and Research, Fundação Hospitalar de Dermatologia Tropical e Venereologia Alfredo da Matta, Manaus, AM, Brazil; b Department of Infectious Diseases, Dermatology, Imaging Diagnosis, and Radiotherapy, Faculty of Medicine, Universidade Estadual Paulista, Botucatu, SP, Brazil; c Graduate Program in Sciences Applied to Dermatology, Amazonas State University, Manaus, AM, Brazil

Dear Editor,

We read with interest the correspondence by Ramírez Portilla, who proposed the concept of a “stigma-induced diagnostic gap” as a critical barrier to leprosy elimination.^1^ This discussion originated from our study evaluating stigma among patients, contacts, and the general population in an endemic region of Brazil,^2^ and we agree that stigma remains one of the most important challenges in leprosy and continues to affect individuals, families, and communities. However, although stigma influences disease perception and healthcare-seeking behavior, delayed diagnosis appears to result from a broader interaction among social, biological, educational, and healthcare-system factors. A recent systematic review and meta-analysis identified multiple determinants of delayed case detection, including low symptom awareness, socioeconomic vulnerability, poor access to healthcare, low educational attainment, diagnostic errors, and stigma-related concerns.^3^

Importantly, the relationship between stigma and delayed diagnosis may be bidirectional. Anticipated discrimination may discourage individuals from seeking medical care, whereas delayed diagnosis increases the likelihood of visible deformities and disabilities, intensifying social exclusion and self-stigmatization. Thus, stigma may function both as a cause and a consequence of diagnostic delay, creating a self-reinforcing cycle within families and communities.^4^ Beyond anticipated discrimination, diagnostic errors, limited access to trained professionals, and the intrinsic clinical complexity of leprosy further contribute to delayed detection. Leprosy has a prolonged incubation period, an indolent clinical course, and early dermatoneurological manifestations that frequently resemble other dermatological and neurological disorders. Consequently, diagnosis may occur only after neural damage has become established.

Therefore, combating stigma must extend beyond educational campaigns. While public awareness is essential, interventions capable of promoting earlier diagnosis are equally necessary. In hyperendemic settings, surveillance strategies aimed at reducing hidden prevalence deserve consideration. One potential framework (Fig. 1) could involve geospatial prioritization of territories with elevated indicators of late diagnosis, structured community-based screening by trained community health agents, targeted risk stratification using immunological or biomolecular tools such as anti-PGL-I IgM serology (ML Flow) or nasal RLEP-PCR, and referral for confirmatory dermatoneurological examination by specialists. Recent evidence suggests that ML Flow may be particularly useful for risk stratification among household contacts and other populations residing in high-transmission settings.^5^

**Figure 1:**
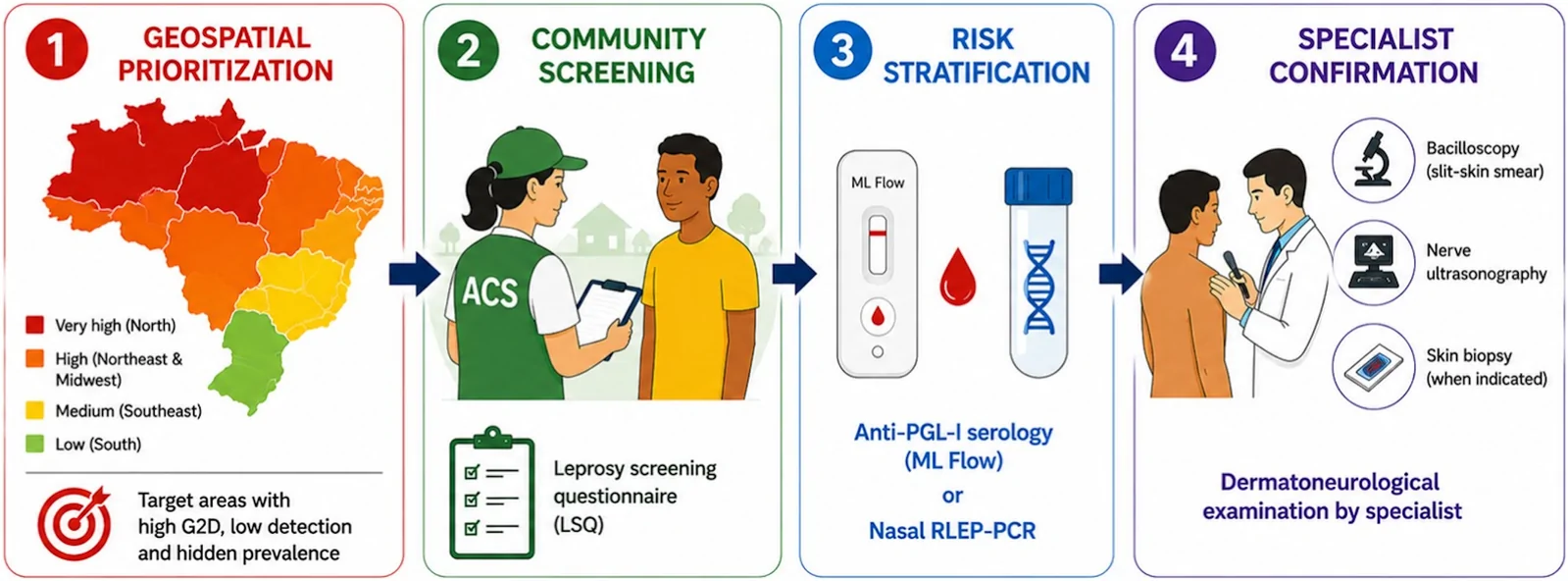
Proposed community framework for early detection of leprosy in an endemic area.

Ultimately, closing the diagnostic gap will require a comprehensive approach that integrates stigma reduction, active case finding, professional training, diagnostic innovation, territorial surveillance, strengthened primary healthcare networks, and action on the biological, epidemiological, and health-system factors that permit ongoing transmission and delayed diagnosis until disability becomes established.

## ORCID ID

Patrícia Tavares Cruz: 0009-0009-6816-585X

Hélio Amante Miot: 0000-0002-2596-9294

Sinésio Talhari: 0000-0001-9753-6706

## Research data availability

Does not apply.

## Financial support

This study was funded by FAPEAM (Fundação de Amparo à Pesquisa do Estado do Amazonas, Brazil) through the *Programa de Apoio à Formação em Ciências Dermatológicas* – PRODERM-RH” (grant nº 010/2023). HAM and ST are recipients of FAPEAM PVN-II research fellowships. PTC received funding from FAPEAM (grant: POSGRAD 002/2023).

## Authors’ contributions

Patrícia Cruz: Study conception and design; data collection; data analysis and interpretation; manuscript drafting; critical literature review; critical manuscript revision; approval of the final version of the manuscript.

Hélio Miot: Study conception and design; Data analysis and interpretation; statistical analysis; manuscript drafting; critical literature review; critical manuscript revision; approval of the final version of the manuscript.

Carolina Talhari: Manuscript drafting; critical literature review; critical manuscript revision; approval of the final version of the manuscript.

Sinésio Talhari: Study conception; manuscript drafting; critical literature review; critical manuscript revision; approval of the final version of the manuscript.

## Conflicts of interest

None declared.

## Declaration of generative artificial intelligence (AI)

During the preparation of this work, the authors used ChatGPT (OpenAI) to assist with English-language editing and readability, and OpenAI’s image generation tool available through ChatGPT Pro to create Fig. 1. The authors reviewed, verified, and edited all AI-assisted outputs, including the figure, to ensure scientific accuracy and consistency with the manuscript content, and take full responsibility for the content of the published article.
